# The potential role of microfilaments in host cells for infection with infectious spleen and kidney necrosis virus infection

**DOI:** 10.1186/1743-422X-10-77

**Published:** 2013-03-07

**Authors:** Kun-tong Jia, Zhao-yu Liu, Chang-jun Guo, Qiong Xia, Shu Mi, Xiao-Dong Li, Shao-ping Weng, Jian-guo He

**Affiliations:** 1 MOE Key Laboratory of Aquatic Product Safety/State Key Laboratory for Biocontrol, School of Marine Sciences, Sun Yat-sen University, 135 Xingang Road West, Guangzhou, 510275, PR China; 2 State Key Laboratory for Biocontrol, School of Life Sciences, Sun Yat-sen University, 135 Xingang Road West, Guangzhou, 510275, PR China

**Keywords:** Iridovirus, Infectious spleen and kidney necrosis virus, Microfilaments

## Abstract

**Background:**

Infectious spleen and kidney necrosis virus (ISKNV) belongs to the genus *Megalocytivirus* from the family Iridoviridae. Megalocytivirus causes severe economic losses to tropical freshwater and marine culture industry in Asian countries and is devastating to the mandarin fish farm industry in China particularly.

**Methods:**

We investigated the involvement of microfilaments in the early and late stages of ISKNV infection in MFF-1 cells by selectively perturbing their architecture using well-characterized inhibitors of actin dynamics. The effect of disruption of actin cytoskeleton on ISKNV infection was evaluated by indirect immunofluorescence analysis or real-time quantitative PCR.

**Results:**

The depolymerization of the actin filaments with cytochalasin D, cytochalasin B, or latrunculin A reduced ISKNV infection. Furthermore, depolymerization of filamentous actin by inhibitors did not inhibit binding of the virus but affected virus internalization in the early stages of infection. In addition, the depolymerization of actin filaments reduced total ISKNV production in the late stages of ISKNV.

**Conclusions:**

This study demonstrated that ISKNV required an intact actin network during infection. The findings will help us to better understand how iridoviruses exploit the cytoskeleton to facilitate their infection and subsequent disease.

## Background

Intracellular pathogens are well known to use and manipulate cellular machinery to accomplish their life cycle. The infection cycle of animal viruses can be divided into three essential steps: entry into a host cell, replication, and egression to ultimately infect another cell. The restrictions of free diffusion in the cytoplasm and the limited coding capacity of viruses force them to manipulate cellular metabolic pathways to achieve each of these steps [1]. Most viruses utilize the cytoskeleton, including actin microfilaments (F-actin) and microtubules, for various stages of their life cycle. The shape of cells, as well as phagocytosis, intercellular communication and the distribution of organelles, depend on actin microfilaments [2]. Microfilaments are the polymers of the protein actin, which exists in monomeric form as globular actin and in filamentous form as filamentous actin [3]. The actin microfilaments often have interesting and surprising roles that are not always well understood. The actin cytoskeleton of the host cell is often co-opted by a virus at different stages of its life cycle to facilitate the infection process [4]. The actin and microtubule cytoskeletons are responsible for the trafficking of numerous endogenous cargos, as well as intracellular microorganisms, such as viruses, throughout the cell [3,5-7]. As obligate intracellular parasites, viruses use the host actin and microtubule transport systems and their motors at every step during their infection cycle, such as attachment, internalization, endocytosis, nuclear targeting, transcription, replication, transport of progeny subviral particles, assembly, exocytosis, and cell-to-cell spread [1,3,4,8,9]. Numerous viral proteins have been reported to interact with actin-binding proteins or directly with actin, such as the baculovirus VP80 protein [10], the NS3 and NS5A proteins (where NS indicates non-structural) of hepatitis C virus [11], the NS1 protein of influenza A [12], and Gag of equine infectious anemia virus [13].

Iridoviruses are large icosahedral cytoplasmic DNA viruses that contain circularly permutated, terminally redundant, double-stranded DNA genomes [14]. The current members of the family *Iridoviridae* are divided into five genera: *Iridovirus*, *Chloriridovirus*, *Ranavirus*, *Lymphocystivirus*, and *Megalocytivirus*[15]. Megalocytiviruses have been implicated in more than 50 fish species infections and currently threaten the aquaculture industry, causing great economic losses in China, Japan, and Southeast Asia [16,17]. Infectious spleen and kidney necrosis virus (ISKNV) is the type species of the genus *Megalocytivirus*, causing severe damage in mandarin fish (*Siniperca chuatsi*) cultures in China [18]. We have previously demonstrated that ISKNV enters mandarin fish fry 1 (MFF-1) cells through a caveola-mediated internalization mechanism, and the microtubules of MFF-1 cells may play a role in the entry of ISKNV [19]. However, involvement of actin filaments in ISKNV infection has not been looked at so far. In the present study, we investigated the involvement of microfilaments in the early and late stages of ISKNV infection in MFF-1 cells by selectively perturbing their architecture using well-characterized pharmacological agents. Our results suggested that the microfilaments played an important role in ISKNV infection.

## Results

### Depolymerization of microfilaments

**Figure 1:**
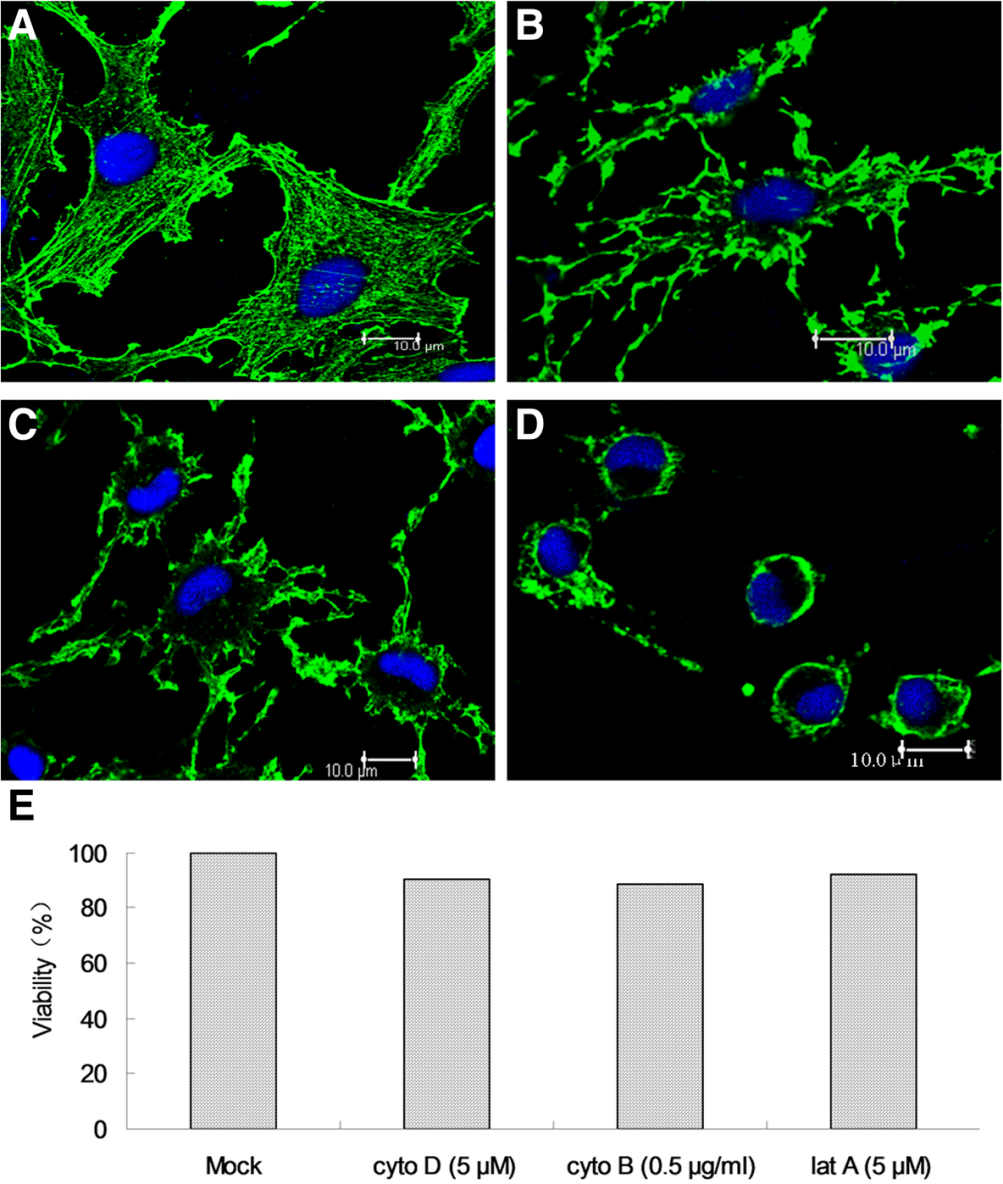
**Effects of drugs on actin microfilaments of MFF-1 cells.** MFF-1 cells were treated for 2 h with 5 μM cyto D (**B**), 0.5 μg/ml cyto B (**C**), or 5 μM lat A (**D**), Mock- treated MFF-1 cells were used as control (**A**). (**E**) Cell viability was measured using the Cell Counting Kit 8 (CCK-8). The scale bar represents 10.0 μm.

We first determined the concentrations of drugs, at which actin microfilaments are disassembled. Cyto D, cyto B and lat A are actin-binding drugs with different modes of action. Lat A binds to monomeric (G)-actin in a 1:1 complex and disrupts polymerization [20]; Cyto D and cyto B bind to F-actin at the barbed ends and disrupts polymerization [21]. When MFF-1 cells were treated with cyto D (5 μM) or cyto B (0.5 μg/ml), the microfilaments in the cytoplasmic region were significantly reduced (Figure 1B and C). Addition of lat A (5 μM) caused the collapse of the cytoplasm and an almost total disappearance of the microfilaments under the membrane (Figure 1D). In contrast, in untreated cells, intact bundles of actin stress fibers spanned the entire cytosol (Figure 1A). These data clearly demonstrate the rapid and specific effects of drugs on microfilament disruption under experimental conditions. The results of cell viability and toxicological tests showed that cell viability was not compromised despite treatment of cells with drugs for as long as 72 h (Figure 1E).

### Effect of disruption of actin cytoskeleton on ISKNV infection

**Figure 2:**
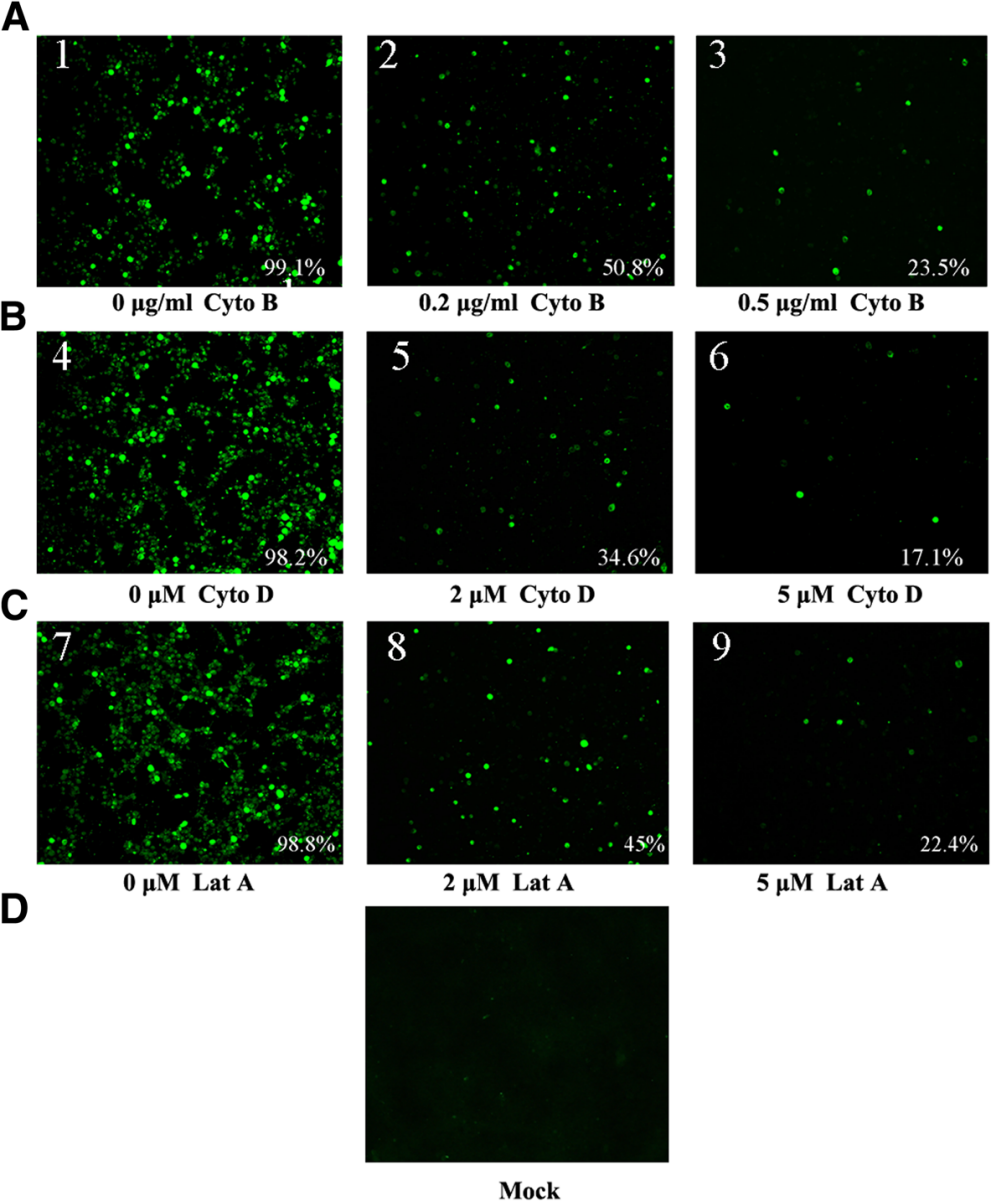
**Effects of disruption of microfilaments on ISKNV infection.** (**A**) MFF-1 cells were treated with 0.2 μg/ml (2) or 0.5 μg/ml cyto B (3), Mock- treated MFF-1 cells were used as control (1). (**B**) MFF-1 cells were treated with 2 μM (5) or 5 μM cyto D (6), Mock- treated MFF-1 cells were used as control (4). (**C**) MFF-1 cells were treated with 2 μM (8) or 5 μM lat A (9), Mock- treated MFF-1 cells were used as control (7) (**D**) Mock-treated and uninfected MFF-1 cells were used as negative control. Numbers at the bottom right of each panel refer to the percentages of infected cells per field and represent the mean of at least four fields of cells from each of three independent experiments. The ISKNV ORF101L expression of infected cells was detected by indirect immunofluorescence (green).

In order to determine if the actin cytoskeleton is required for ISKNV infection, we treated MFF-1 cells with a panel of chemical inhibitors at a concentration determined by the above experiments. Cells were fixed and examined for the expression of ISKNV ORF101L protein, a viral structural protein, by immunofluorescence 48 h post-infection (hpi) (Figure 2, panels 1, 4, and 7). As shown in Figure 2A, the infection rates of ISKNV were 50.8% and 23.5% in the presence of 0.2 and 0.5 μg/ml of cyto B, respectively, which were significantly smaller than the infection rates of the positive control (99.1%). A similar situation was detected in cells treated with cyto D or lat A. The infection rates of ISKNV were 34.6% and 17.1% in the presence of 2 μM and 5 μM of cyto D, respectively, which were significantly smaller than the infection rates of the positive control (98.2%) (Figure 2B). The infection rates of ISKNV were 45% and 22.4% in the presence of 2 μM and 5 μM of lat A, respectively, which were smaller than the infection rates of the positive control (98.8%) (Figure 2C). Untreated and uninfected cells served as negative control (Figure 2D).

### Effects of actin filaments on early stages of ISKNV infection

**Figure 3:**
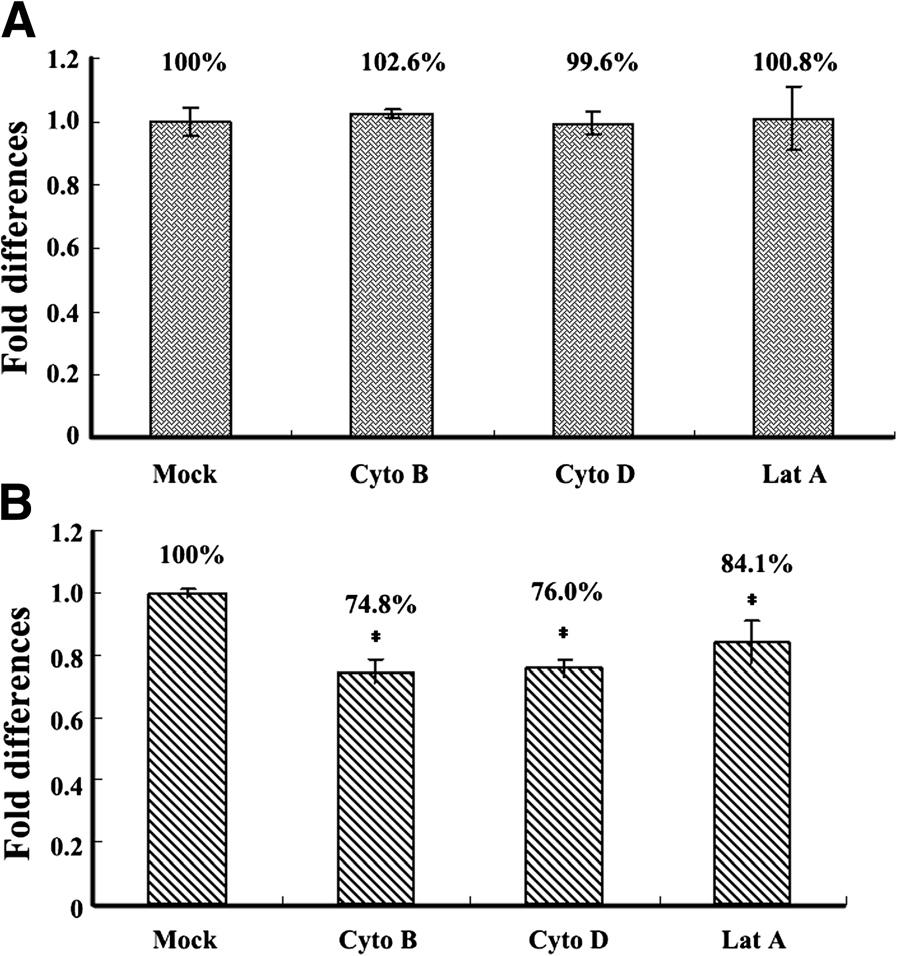
**Intact actin filaments are required for virus internalization.** (**A**) Effects of disruption of microfilaments on ISKNV binding; (**B**) Effects of disruption of microfilaments on ISKNV internalization. All samples were subjected to qPCR analysis and the levels of MCP DNA relative to those of actin were determined and are represented as fold DNA change relative to control (Mock). The asterisks indicate that the difference between the mean value is statistically significant (P<0.05) compared to the untreated control.

Because the preceding experiments in this work showed that depolymerization of actin microfilaments caused a significant decrease in the expression of ISKNV ORF101L, we performed a number of experiments to investigate the role of microfilaments in early ISKNV infection. Results showed that ISKNV DNA levels were similar in control, cyto B, cyto D and lat A treated cells (Figure 3A), suggesting that depolymerization of actin microfilaments did not affected binding of ISKNV to MFF-1 cells.

Internalization of virus was measured in the presence of cyto B, cyto D or lat A just as described in the materials and methods. The relative amount of viral DNA in each treatment indicated the number of virus particles that had entered the cells. Data analysis showed that ISKNV DNA levels were reduced in cyto B (74.8%), cyto D (76%) and lat A (84.1%) treated cells compared with control cells (Figure 3).

### Effects of actin filament depolymerization on late stages of ISKNV infection

**Figure 4:**
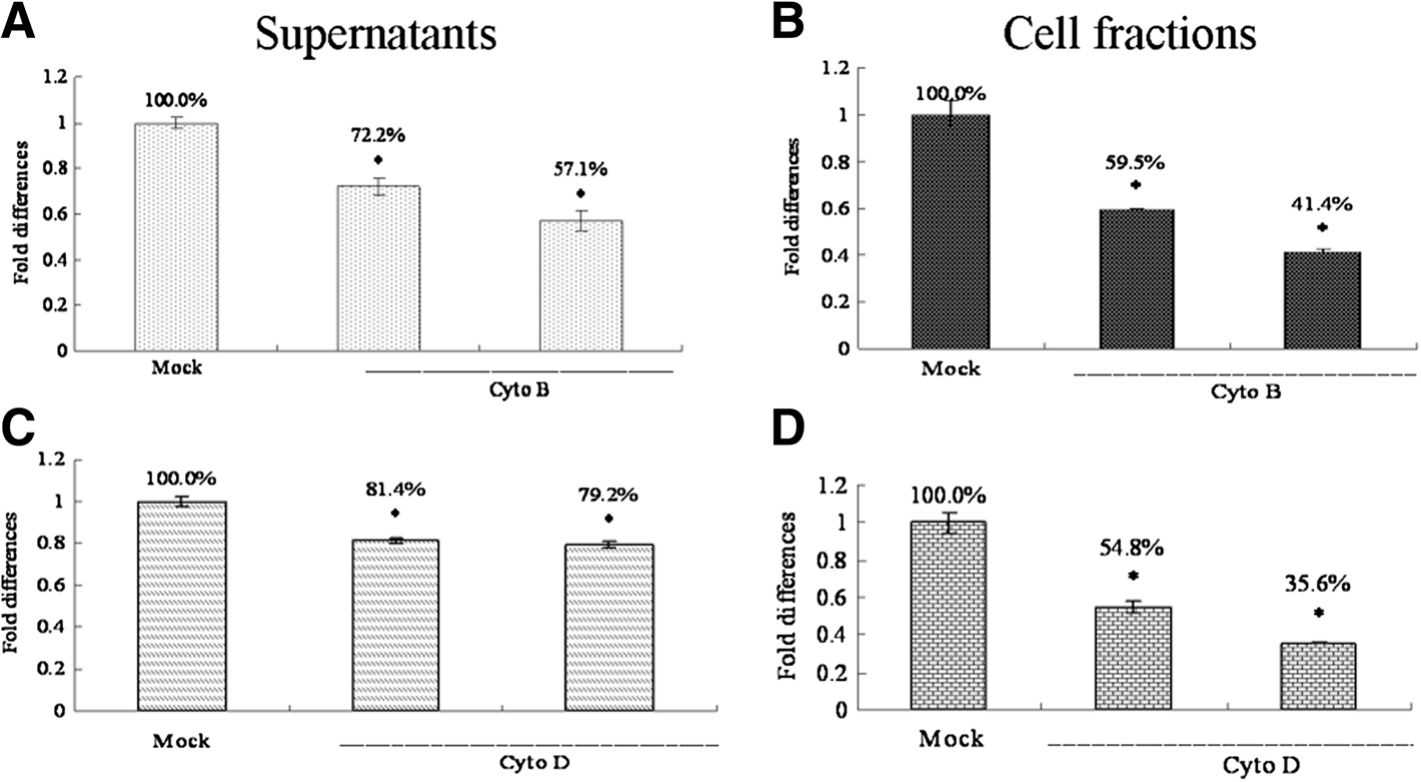
**Effects of actin depolymerization on late stages of ISKNV infection.** Infected MFF-1 cells were incubated with increasing concentrations of cyto B (**A, B**) or cyto D (**C, D**) for 72 h at 27°C. Each experiment was repeated independently at least three times. The data represent the mean value with the standard deviation of the reduplicates. The asterisks indicate that the difference between the mean value is statistically significant (P<0.05) compared to the untreated control (Mock).

To evaluate further the involvement of the actin microfilaments in the viral life cycle steps after entry, ISKNV-infected MFF-1 cells were incubated with different concentrations of inhibitors. To differentiate between effects on distinct viral processes, we performed the experiment as described in the materials and methods. Results showed that ISKNV production was decreased for cyto B and cyto D treated cells compared to control (Figure 4). Virus collected from the supernatants was reduced by cyto B incubation in a dose-dependent manner with a 42.9% reduction at 0.5 μg/ml of cyto B compared with that in untreated cells (Figure 4A). To determine whether the reduced viral budding induced by cyto B treatment was a common effect of actin filament-disrupting drugs, we also tested cyto D, another reagent that specifically depolymerizes actin filaments. Similarly, a 20.8% decrease in virion production was detected in the supernatants of cells treated with this compound (5 μM) (Figure 4C).

We also examined the amount of virus present in the cell-associated fraction from these samples. The results showed that the inhibitors caused a great reduction in viral growth in the cell-associated fraction. Treatment with the inhibitors resulted in inhibition of viral DNA by approximately 58.6% and 64.6% for cyto B and cyto D, respectively, compared with the control (Figure 4B and D). To determine the effect of the total mount of virus, we summed the intracellular and extracellular viruses in each mock or drug treated samples. In drug treated cells virus levels remained significantly (p<0.05) lower, suggesting that there was less virus overall (data not shown).

## Discussion

Many viruses have been reported to exploit the host cellular machinery throughout their life cycle due to their parasitic nature and simplicity [22]. Several reports showed that the cytoskeleton plays an important role in the intracellular traffic of some viruses [9,23-26]. Frog virus 3 (FV3) was found to interact with the cytoskeleton and disrupt the actin cytoskeleton at the initial stages of infection [27]. Treatment of infected cells with cytochalasin has been demonstrated to affect the release of FV3 at the plasma membrane level. Tiger frog virus (TFV) was reported to cause the reorganization of microtubules in infected zebrafish embryo fibroblast 4 (ZF4) cells [28].

In the present study, we found that depolymerization of the actin filaments with cyto D, cyto B, or lat A reduced ISKNV infection, the virus blockage at the entry step of its life cycle potentially caused the reduced ISKNV infection. In addition, the depolymerization of actin filaments reduced both the total amount of virus produced in the cell and the amount of virus that was allowed to egress from cells in the late stages of ISKNV infection. These data demonstrate that ISKNV relies on an intact actin network during infection.

Increasing evidence has showed that the actin cytoskeleton is involved in many endocytic pathways, although to varying degrees [29]. Entry by endocytosis may require remodeling of the actin cytoskeleton, while fusion at the cell surface might not rely as heavily on the actin cytoskeleton [28]. Our results showed that microfilament depolymerization did not change virus binding to the cell, but it efficiently inhibited virus internalization. Many previous reports have demonstrated that microfilaments are dispensable for viral binding to the host cell [30-32]. The role of microfilaments in viral internalization may be useful to better understand the precise entry mechanism of ISKNV.

Actin filaments have been shown to be essential for infection by several other viruses [23,33,34]. Using inhibitor depolymerizing actin filaments, we evaluated the effect of disrupting actin systems on the infectivity of ISKNV. Our results indicated that disruption of microfilaments with cyto D, cyto B, or lat A inhibited the infection of MFF-1 cells by ISKNV. Furthermore, using qPCR, we found that disrupting microfilaments inhibited early steps of virus entry. However, the disruption of microfilaments could not inhibit the virus entry completely, which could be attributed to a caveola-mediated internalization mechanism through which ISKNV enters MFF-1 cells. Similar to other viruses, ISKNV might use more than one route to enter cells. In this case, inhibition of one pathway might not affect viral entry via another pathway, resulting in a reduced number of viral particles entering the cells [35]. In fact, cells have been demonstrated to upregulate alternate endocytic routes if an endocytic pathway is blocked [36]. Moreover, caveolae and caveolin-associated signaling proteins and receptors have been reported to be linked to a dynamic filamentous actin network via structural proteins [37]. The disruption of actin may destroy the caveola-mediated internalization mechanism through which ISKNV enters MFF-1 cells and then impede ISKNV infection. Further studies are needed to clarify the role of actin in caveola-mediated endocytosis during ISKNV entry and trafficking in MFF-1 cells.

We also sought to determine the effect of inhibitors on later stages of viral replication. In the present study, we evaluated the replication ability of ISKNV in presence of actin inhibitors and found a significant reduction in virus replication. These results indicate that the microfilaments are possibly involved in an interaction with the viral replication machinery. Several reports have shown that actin microfilaments participate in late stages of viral replication, such as assembly and release [38]. Treatment with the cyto D, the *Autographa californica* nucleopolyhedrovirus budding from host cells was drastically inhibited [39]. Cyto D caused numerous microvillus-like projections containing virions and actin microfilaments to accumulate on the infected cell surface in the late stage of frog virus 3 infections [27]. The utilization of a cellular cytoarchitecture for viral replication has also been reported in several viruses, such as human parainfluenza virus type 3 [40], mouse mammary tumor virus [41], and measles virus [42]. To date, little is known about the accurate kinetics of ISKNV replication cycle. Our results showed that treatment with cyto D and cyto B reduced total ISKNV production (Figure 4), but which late step(s) of the viral life was affected by microfilaments should be further studies. All these results suggested that actin filaments played an important role in viral replication cycle in vitro using the MFF-1 cell line.

In addition, many viruses may employ the actin and microtubule network to transport their nucleocapsids protein [43]. Nucleocapsids of the murine mammary tumor virus have been found to interact with actin with this interaction reported to be necessary for extruding virus particles from infected cells [44]. Xiong et al. (2011) suggested that the ISKNV major capsid protein (MCP) gene interacts with the β-actin of zebrafish. In our study, we also find that the actin of MFF-1 cells interacts with the MCP of ISKNV by co-immunoprecipitation (data not shown). All the results provide strong evidence that the actin network potentially participates in ISKNV intracellular traffic and the release of virus from cells.

## Conclusions

In summary, we have studied the roles of actin filaments in ISKNV infection, and found that they played an important role in the entry into MFF-1 cells and later stages of ISKNV replication cycle.

## Materials and methods

### Cells and virus

MFF-1 cells were maintained in Dulbecco’s modified Eagle’s medium (DMEM) (Gibco, USA) supplemented with 10% (v/v) fetal bovine serum (Gibco, USA) and passaged every 3–4 days by trypsinization, in a monolayer at 27°C, in a humidified atmosphere with 5% CO_2_. The ISKNV (ISKNV strain NH060831) used in this study was originally isolated from diseased mandarin fish and maintained by our laboratory.

### Antibodies and reagents

The rabbit polyclonal anti-ORF101L antisera used in this study was generated previously by our laboratory [45]. Alexa Fluor®488-labeled goat anti-mouse IgG, Alexa Fluor®488-labeled anti-rabbit secondary antibody and Hoechst 33342 were purchased from Invitrogen (Eugene, OR, USA). Cytochalasin D, cytochalasin B and latrunculin A were obtained from Sigma-Aldrich (St. Louis, MO, USA). Cytochalasin D was reconstituted in DMSO to a concentration of 100 μM and stored at -20°C. Cytochalasin B was reconstituted in DMSO to a concentration of 10 μg/ml and stored at -20°C. Latrunculin A was reconstituted in DMSO to a concentration of 100 μM and stored at -20°C.

### Cell viability assay

Cell viability and toxicological tests with inhibitors were performed as previously described, using Cell Counting Kit 8 (CCK-8) [19].

### Depolymerization of microfilaments

MFF-1 cells were grown to 70% confluence on cover slips. Collapse of the actin filaments was achieved by treating MFF-1 cells with 5 μM lat A, 5 μM cyto D, 0.5 μg/ml of cyto B or solvent only for 2 h at 27°C. Following either mock treatment or a given cytoskeleton treatment, the cells were fixed and stained to evaluate the action of the corresponding drug.

Treated MFF-1 cells were washed three times in phosphate-buffered saline (PBS) and fixed in 4% paraformaldehyde for 10 min to visualize the actin filaments. Ten minutes of permeabilization in 1% Triton X-100 was followed by a 30 min blocking step in 5% goat serum to reduce non-specific binding. The cells were then incubated with 1:100 dilution of mouse anti-actin antibody (Abcam, USA) for 1 h at 37°C. After three washes in PBS, the primary antibody was recognized by a secondary goat anti-mouse Alexa Fluor®488-labeled antibody used at 1:300 dilution for 1 h at 37°C. The cells were washed and mounted on glass slides with Hoechst 33342. Samples were viewed and evaluated under a confocal microscope (Zeiss LSM510) equipped with 555/488 nm argon/krypton and 543 nm helium/neon lasers.

### Indirect immunofluorescence analysis of ISKNV infection

ISKNV-infected MFF-1 cells were fixed in 4% paraformaldehyde after 48 hpi to detect the expression of ISKNV ORF101L. Cells were washed three times with PBS and permeabilized with 1% Triton X-100 in PBS for 10 min. Cells were rinsed three times with PBS, and non-specific binding was reduced by blocking with 5% goat serum for 30 min at RT. Cells were incubated with anti-ORF101L antibody and in PBST (PBS plus 0.05% Tween-20) containing 5% goat serum for 60 min at RT. Cells were rinsed three times for 10 min with PBST and incubated with Alexa Fluor®488-labeled anti-rabbit secondary antibody at a dilution of 1:1000 for 1 h. The cover slips were then washed several times with PBST and mounted with Hoechst 33342. Samples were viewed and evaluated under a confocal microscope (Zeiss LSM510) equipped with 555/488 nm argon/krypton and 543 nm helium/neon lasers.

### Measurement of virus binding and internalization

For virus binding assays, MFF-1 cells were grown on 6-well plates overnight to achieve 70–80% confluency and then pretreated with cyto B, cyto D or lat A for 2 h at 27°C. The cells were then inoculated with ISKNV at a multiplicity of infection (MOI) of 10 in the presence of the inhibitors at 4°C for 1 h. After washed three times with PBS, DNA was isolated using E.Z.N.A.®Tissue DNA Kit (Omega, USA) and the number of virus copies bound cell was determined by qPCR. To assess internalization, cells were pretreated similar to the binding assay above, and then ISKNV internalization was allowed to proceed for 2 h at 27°C in the presence of the inhibitors. At the end of the incubation period, cells were treated with 1 mg/ml of proteinase K (Roche) in PBS with 10 mM EDTA for 10 min to remove virus remaining at the cell surface. Total DNA of cell pellets was isolated for qPCR.

### Effect of disruption of actin cytoskeleton on ISKNV infection

MFF-1 cells grown on 24-well plates at 80% to 90% confluence were preincubated with lat A, cyto D, or cyto B at different concentrations for 2 h at 27°C before infection. Their appropriate concentrations were determined by titration (data not shown). Pretreated and untreated MFF-1 cells were challenged with the virus at an MOI of 10 in the continued presence or absence of these drugs for 4 h at 27°C, after which the virus inoculum was removed. After cells were washed once with PBS, treated cells were incubated with medium containing inhibitors and untreated cells were incubated with normal medium for 48 h at 27°C. Cells were fixed 48 hpi and stained for ISKNV ORF101L expression as described above.

### Production of budded virus in the presence of actin filament inhibitors

In an assay to evaluate the production of budded virus in the presence of actin filament inhibitors, MFF-1 cells were grown on 24-well plates at 80% to 90% confluence and incubated with the ISKNV at an MOI of 10 for 4 h at 27°C. The virus inoculum was then removed, and the cells were washed gently twice with fresh medium. Each well were incubated with 500 μl of fresh medium with or without different concentrations of cyto B or cyto D at 27°C. This medium was sampled 72 hpi. All samples were frozen at −80°C immediately after they were taken. Virion production was measured by absolute real-time qPCR. Each experiment was performed twice independently.

### Real-time qPCR

ISKNV-infected cells were incubated with different concentrations of the inhibitors for 72 h at 27°C, and the supernatants and cell fractions were collected. Viral DNA of the supernatants was extracted to analyze the inhibition of release of virus by the compounds using Purelink Viral RNA/DNA Mini Kit (Invitrogen) as recommended by the manufacturer. The level of ISKNV GEs was determined by absolute real-time qPCR using LightCycler 480 (Roche, Germany). Briefly, reactions were performed in a 10 ml volume containing 2 ml of total DNA, 5 ml of 2 × SYBR® Premix Ex Taq™ (TaKaRa, China), 0.2 μl of ISKNV MCP-specific forward primer (5^′^-CAATGTAGCACCCGCACTGACC-^′^3), and 0.2 μl of reverse primer (5^′^-ACCTCACGCTCCTCGCTTGTC- ^′^3). A pCMV-myc-MCP vector containing one copy of the ISKNV MCP gene was serially diluted 10-fold (10^11^ to 10^4^ copies) and used in parallel as a standard. The cycling parameters were as follows: one cycle of 95°C for 30 s and 40 cycles of 95°C for 5 s, 60°C for 20 s, and 70°C for 20 s, followed by one cycle of 95°C at 5°C/s calefactive velocity to generate the melting curve. Fluorescence measurements were taken at 70°C for 0.1 s during each cycle. A standard curve of the Ct, based on known amounts of plasmid DNA containing the MCP gene, was determined by linear regression analysis. The number of viral DNA molecules was then calculated by using the equation of the straight line. *p*<0.05 was considered statistically significant, and the data were expressed as mean ± standard deviation.

Total DNA of cell fractions was extracted for qPCR analysis to analyze the inhibition of viral replication by the compounds using E.Z.N.A.®Tissue DNA Kit (Omega, USA) according to the manufacturer’s instructions. Samples were analyzed by qPCR using the following oligonucleotides as described above: MCP-specific forward primer, 5^′^-CAATGTAGCACCCGCACTGACC-3^′^; MCP-specific reverse primer, 5‘-ACCTCACGCTCCTCGCTTGTC-3’; Actin-F, 5‘-CCCTCTGAACCCCAAAGCCA-3’; and Actin-R, 5‘-CAGCCTGGATGGCAACGTACA-3’.

Inhibitor-treated MFF-1 cells were infected with ISKNV at an MOI of 10 to analyze the inhibition of virus entry by the compounds. At 4 hpi, total DNA was extracted for qPCR analysis using E.Z.N.A.®Tissue DNA Kit according to the manufacturer’s instructions. Samples were analyzed by qPCR as described above. The C_T_ values for the cellular control gene actin were subtracted from the DNA C_T_ values, and the result was designated as ΔC_T_. The average ΔC_T_ value was determined for triplicate samples, and the numbers for each inhibitor were compared with the ΔC_T_ values for solvent alone. The difference in changes between virus DNA levels with the inhibitors and those without was calculated by subtracting the ΔC_T_ of the sample treated with solvent alone from the ΔC_T_ value in the presence of each of the inhibitors, which yielded a ΔΔC_T_ value. For ease of interpretation, these values were converted into fold differences using the equation of 2^−ΔΔCT^.

## Abbreviations

ISKNV: Infectious spleen and kidney necrosis virus; MFF-1: Mandarin fish fry 1; Cyto D: Cytochalasin D; Cyto B: Cytochalasin B; Lat A: Latrunculin A; F-actin: Actin microfilaments; DMEM: Dulbecco’s modified Eagle’s medium; P.i.: Post-infection; TFV: Tiger frog virus; FV3: Frog virus 3; ZF4: Zebrafish embryo fibroblast 4; MOI: Multiplicity of infection; MCP: Major capsid protein; QPCR: Real-time PCR

## Competing interests

The authors declare that they have no competing interests.

## Authors’ contributions

KJ participated in all the laboratory studies and prepared the manuscript; ZL and QX helped KJ in the analysis of qPCR results; SM worked on the cell viability and toxicological tests; CG read the manuscript and helped KJ in manuscript writing and publishing; SW and LX removed the language problems; JH is the PhD supervisor of KJ. All authors read and approved the final manuscript.
